# EMOTION BELIEFS AND DAILY AFFECTIVE EXPERIENCES ACROSS THE ADULT LIFESPAN

**DOI:** 10.1093/geroni/igae098.2109

**Published:** 2024-12-31

**Authors:** Jocelyn Rutledge

**Affiliations:** Wilfrid Laurier University, Waterloo, Ontario, Canada

## Abstract

Emotion beliefs refer to the extent to which people believe emotions “should” and “can” be controlled. Importantly, past research has linked peoples’ emotion beliefs to the extent to which they utilize effective emotional regulation strategies, and psychological well-being. To our knowledge, research has yet to examine the association between emotional beliefs and affective experiences at the daily-level, and how developmental factors are associated with emotion control beliefs in adults. The present research sought to examine age differences in within-person associations of daily emotion beliefs and daily positive and negative affect. To do so, we used 14-day daily diary data from an adult lifespan community sample [N = 91, age range = 19-92]. Multilevel models revealed that when younger (but not older) people endorsed higher-than-normal beliefs they “should” control their emotions they reported greater negative affect, while higher-than-normal beliefs they “can” control their emotions were associated with higher positive affect. In addition, when older (but not younger) people endorsed higher-than-normal beliefs that they “can” control their emotions they experienced greater declines in negative affect. These findings add to theory and research on emotional aging by highlighting that: 1) emotion beliefs are differentially associated with affective experience on a daily-level, and 2) these associations change across the adult lifespan.

